# The impact of the absence of aliphatic glucosinolates on water transport under salt stress in *Arabidopsis thaliana*

**DOI:** 10.3389/fpls.2015.00524

**Published:** 2015-07-15

**Authors:** Mcarmen Martínez-Ballesta, Diego A. Moreno-Fernández, Diego Castejón, Cristina Ochando, Piero A. Morandini, Micaela Carvajal

**Affiliations:** ^1^Plant Nutrition Department, Centro de Edafología y Biología Aplicada del Segura (CEBAS-CSIC)Murcia, Spain; ^2^Food Science and Technology Department, Centro de Edafología y Biología Aplicada del Segura (CEBAS-CSIC)Murcia, Spain; ^3^Department of Biosciences, CNR Biophysics Institute, University of MilanMilano, Italy

**Keywords:** *Arabidopsis thaliana*, Brassicaceae, glucosinolates, hydraulic conductance, plasma membrane intrinsic protein

## Abstract

Members of the Brassicaceae are known for their contents of nutrients and health-promoting phytochemicals, including glucosinolates. Exposure to salinity increases the levels of several of these compounds, but their role in abiotic stress response is unclear. The effect of aliphatic glucosinolates on plant water balance and growth under salt stress, involving aquaporins, was investigated by means of *Arabidopsis thaliana* mutants impaired in aliphatic glucosinolate biosynthesis, which is controlled by two transcription factors: Myb28 and Myb29. The double mutant *myb28myb29*, completely lacking aliphatic glucosinolates, was compared to wild type Col-0 (WT) and the single mutant *myb28*. A greater reduction in the hydraulic conductivity of *myb28myb29* was observed under salt stress, when compared to the WT and *myb28*; this correlated with the abundance of both PIP1 and PIP2 aquaporin subfamilies. Also, changes in root architecture in response to salinity were genotype dependent. Treatment with NaCl altered glucosinolates biosynthesis in a similar way in WT and the single mutant and differently in the double mutant. The results indicate that short-chain aliphatic glucosinolates may contribute to water saving under salt stress.

## Introduction

Glucosinolates are a group of plant secondary metabolites containing nitrogen and sulfur, mainly found in the Brassicaceae family (Zareba and Serradelf, 2004; Finley, 2005). Based on their side-chain structure and amino acid precursors, glucosinolates can be classified into aliphatic, aromatic, and indolic (Fahey et al., 2001). In *Arabidopsis thaliana* (Arabidopsis), nearly 40 different glucosinolates have been identified, mostly of the aliphatic or indolic type (Reichelt et al., 2002; Brown et al., 2003).

It has been reported that two MYB transcription factors, MYB28 and MYB29, were essential for aliphatic glucosinolate biosynthesis (Hirai et al., 2007; Gigolashvili et al., 2007b; Sonderby et al., 2007; Beekwilder et al., 2008; Malitsky et al., 2008). Thus, the levels of short-chain aliphatic glucosinolates are roughly halved in quantity in both the myb28 and myb29 single mutants in *A. thaliana*, but long-chain ones are absent in the myb28 mutant. The *MYB29* gene plays an additional function in the methyl jasmonate-mediated induction of aliphatic glucosinolate biosynthetic genes (Hirai et al., 2007; Sonderby et al., 2007; Beekwilder et al., 2008; Gigolashvili et al., 2009).

The role of glucosinolates in plant defense against herbivorous insects is well established. After tissue damage, myrosinase activity hydrolyses glucosinolates, providing reactive compounds active against insects and pathogens (Beekwilder et al., 2008; Kim et al., 2008; Schlaeppi and Mauch, 2010). For instance, aliphatic glucosinolates play an important role in plant-herbivore interactions (Beekwilder et al., 2008; Redovnikovic et al., 2008) and in non-host resistance in the Arabidopsis-Pseudomonas system (Fan et al., 2011). However, in Arabidopsis, the suppression of aliphatic glucosinolates by RNA interference (RNAi) was also found to affect proteins and metabolites involved in photosynthesis, oxidative stress and hormone metabolism (Chen et al., 2012) as well as the circadian rhythms influences the expression of glucosinolates biosynthetic genes and other abiotic stress response-related genes (Rosa, 1997), highlighting the complexity of glucosinolate function in plants.

Thus, many abiotic factors have been reported to affect the glucosinolate profile and content (Schonhof et al., 2007; Haugen et al., 2008; Kuhlmann and Muller, 2009; Schreiner et al., 2009; Martínez-Ballesta et al., 2013), including salt stress—whose effects on glucosinolate accumulation have been investigated in *Brassica* species and *Raphanus sativus* (Qasim et al., 2003; Ashraf and Haris, 2004; Lopez-Berenguer et al., 2008; Yuan et al., 2010). However, little is known about the role of different glucosinolates - as well as their hydrolysis products, isothiocyanates—in abiotic stress responses. In this context, exogenous application of allyl-isothiocyanate induced stomatal closure in Arabidopsis through the inhibition of inward K^+^ channels present in the guard cells, allowing water conservation and preventing pathogen attack under water stress (Khokon et al., 2011; Li et al., 2014). Similarly, isothiocyanates induced heat tolerance in Arabidopsis plants by increasing the expression levels of heat shock proteins (HSP) (Hara et al., 2013). Administration of, sinigrin, an aliphatic glucosinolate, regulated water transport by increasing both movement through the symplastic water pathway and aquaporin abundance (Martínez-Ballesta et al., 2013).

The fact that moderate salt stress increases glucosinolate levels (Qasim et al., 2003; López-Berenguer et al., 2008; Keling and Zhujun, 2010) suggests that, under low water potential, these secondary metabolites could be an adaptive component of salt tolerance in members of the Brassicaceae family (Stroeher et al., 1995).

On the other hand, there are contrasting results concerning the relationship between glucosinolates biosynthesis and hormone-mediated plant growth and development. For instance, a mutation in the CYP83B1 (*SUR2*) and C-S lyase (*SUR1*) genes in *A. thaliana*, which are involved in the glucosinolates pathway, resulted in adventitious roots development—mediated by an increase in the levels of auxin (indole-3-acetic acid, IAA) (Smolen and Bender, 2002). Similarly, overexpression of the ATR1/MYB34 and MYB122 transcription factors led to increased levels of glucosinolates and high-IAA phenotypes. However, overexpression of HIG1/MYB51, another MYB family member involved in the regulation of the indole glucosinolate biosynthesis, did not produce any aberrant growth phenotypes (Gigolashvili et al., 2008).

We have explored the role of endogenous glucosinolate accumulation in the plant response to salinity, in relation to water uptake and transport, as well as plant morphological changes to cope with this stress. To this end we examined the phenotypic changes and glucosinolate profiles of the different genotypes as well as their ability to take up and transport water, the aquaporin abundance and modifications of root architecture under salt stress.

## Materials and methods

### Plant material and growth conditions

Plants of *A. thaliana* ecotype Col-0 wild type (WT), the *myb28* single mutant and the *myb28myb29* double mutant, previously described (Beekwilder et al., 2008), were used throughout this work. The single mutant *myb28* line had reduced levels of short-chain aliphatic glucosinolates whereas long-chain aliphatic glucosinolates are absent. The single mutant *myb29* line showed reduced accumulation of short-chain glucosinolates while the levels of long-chain are similar to the the wild type; however, it has been observed that there was no modification of the expression of glucosinolates biosynthetic genes by suppression of this transcription factor (Hirai et al., 2007), indicating that—rather than being essential for glucosinolates synthesis—*myb29* participates in glucosinolates induction. The *myb28myb29* double mutant knockouts decrease the accumulation of both short- and long-chain aliphatic glucosinolates to undetectable levels. In order to distinguish the effects of short- and long-chain glucosinolates on water relations under salinity, both the *myb28* single and the double *myb28myb29* mutants were employed in this work.

Seeds were surface-sterilized for 30 s in 70% ethanol, followed by 10% (v/v) commercial bleach for 10 min. They were then washed with sterilized water four times and suspended in sterile water at 4°C for 72 h. Plants (64) of each genotype and treatment were grown in hydroponic culture (Gibeaut et al., 1997) in a controlled-environment chamber (8h/16h day/night cycle, 150 mmol m^−2^ s^−1^ light, 22°C). The plants were grown in a modified, one-fifth-strength Hoagland solution with the following macronutrients (mM): 1.4 Ca(NO_3_)_2_, 0.35 MgSO_4_ and 0.1 Ca(H_2_PO_4_)_2_ and the following micronutrients (μM): 50 CaCl_2_, 12.5 H_3_BO_3_, 1 MnSO_4_, 1 ZnSO_4_, 0.5 CuSO_4_, 0.1 H_2_MoO_4_, 0.1 NiSO_4_, and 10 Fe-EDDHA. For each experiment, as indicated, K^+^ was added as KCl. The pH was adjusted daily to 5.5 and the solutions were renewed weekly.

The plants were allowed to grow for 4 weeks and then NaCl was added to the nutrient solution of half of the plants of each genotype, to give a final concentration of 100 mM. After 10 d, the plants were collected for analysis.

### Analysis of primary and lateral root length and leaf area determination

The primary root length and lateral roots (LRs) length were determined using an optical microscope system with a computer attachment (MPS 60, Leica, Wetzlar, Germany). Root lengths were measured on digital images using ImageJ 1.40 software (http://rsb.info.nih.gov/ij/), from the analysis of 20 plants. The experiments were performed at least twice, independently. Leaf area was determined using an area meter (model LI-3100C; Li-Cor, Lincoln, NE, USA).

### Measurement of fresh and dry weights and relative water content

Twenty plants per treatment were chosen for the determination of fresh weight (FW), which was directly recorded with a portable balance (Scout Pro 400 g, Ohaus Corporation, NJ, USA). The plants were dried in an oven at 70°C until constant weight, to determine the dry weight (DW). In order to determine the leaf relative water content (RWC), five discs of 1-cm diameter were sampled. The fresh mass (Mf) of the discs was measured immediately; then, they were hydrated for 24 h at 4°C, in the dark, to determine the fully-turgid mass (Mt). After this, the discs were dried at 70°C, to obtain their dry mass (Md). The RWC was calculated as: (Mf - Md)/(Mt - Md).

### Water relations

The root hydraulic conductance (L_0_) of the Arabidopsis plants was measured by pressurizing the roots in a Scholander pressure chamber, as described in Javot et al. (2003). The aerial parts of the plant were removed and the freshly-excised roots were inserted into the pressure chamber, in a plastic tube with the same nutrient solution used for their growth. A gradual increase of pressure (from 0.1 to 0.4 MPa) was applied to the detached roots. The sap that accumulated in this pressure range during a certain time, according to the treatment, was collected in a graduated glass micropipette. The sap flow (Jv) was expressed in mg g^−1^ (root dry weight) h^−1^ and plotted against pressure (MPa), the slope being the L_0_ value in mg g^−1^ (root dry weight) h^−1^ MPa^−1^. The measurements were made in the middle of the photoperiod, 10 d after applying NaCl. Data were obtained from 12 measurements for each genotype.

Whole plant transpiration was determined by weight loss of each pot (sealed in a plastic tube at the base of the stem) in a 5-h daytime period, divided by the total leaf area at harvest, expressed in units of mg m^−2^ (leaf area) h^−1^.

### Mineral elements

The macronutrients (calcium, Ca; potassium, K; magnesium, Mg; sodium, Na; phosphorus, P; and sulfur, S) were analyzed in oven-dried leaf tissue (ca. 100 mg DW). The samples were digested, after HNO_3_-H_2_O_2_ (2:1) addition, in a microwave oven (CEM Mars Xpress, North Carolina, USA) and analyzed by ICP spectrometry (Iris Intrepid II, Thermo Electron Corporation, Franklin, USA). Also, the concentrations of C and N were determined in samples (ca. 2–3 mg DW) of leaves, using a CN analyzer (Thermo-Finnigan 1112 EA elemental analyzer; Thermo-Finnigan, Milan, Italy).

### Extraction and determination of intact glucosinolates and phenolic compounds

A standard procedure (Bennett et al., 2004) was used for the extraction of intact glucosinolates with modifications. For this, freeze-dried leaf powder (150 mg) was extracted with 1.5 mL of 70% methanol (MeOH); heated at 70°C for 30 min, shaking every 5 min with a vortex stirrer, followed by centrifugation (30 min, 13,000 *g*, 4°C) to pellet insoluble material. The supernatants were collected and the methanol was removed using a rotary evaporator; the dry material obtained was re-dissolved in the same volume of ultrapure water, as the original supernatant, and filtered through a 0.45-μm polyethersulphone filter (ANOTOP 10 plus; Whatman, Maidstone, UK). For glucosinolate analysis, each sample (20 μL) was analyzed by a Waters HPLC system (Waters Cromatografía S.A., Barcelona, Spain) consisting of a W600E multi-solvent delivery system, in-line degasser, W717plus Autosampler and W2996 Photodiode. The compounds were separated in a Luna C18 column (25 × 0.46 cm, 5 μm particle size; Phenomenex, Macclesfield, UK) with a security guard C18-ODS (4 × 30 mm) cartridge system (Phenomenex). The mobile phase was a mixture of water/formic acid (99:1 v/v) (A) and acetonitrile (B). Glucosinolates were eluted off the column in 35 min. The flow rate was 1 mL min^−1^ in a linear gradient, starting with 1% B and reaching 20% B in 30 min and 1% B in 40 min. Glucosinolates present in the samples were then identified using a previously-described LC-MS method (Martínez-Sánchez et al., 2006) and quantified by HPLC-DAD, using sinigrin (sinigrin monohydrate from *Sinapis nigra*, Phytoplan Diehm & Neuberger GmbH, Heidelberg, Germany) as standard. The glucosinolate concentration was expressed as mg per 100 g of fresh weight.

The chromatograms of phenolic compounds were recorded at 330 nm and quantified using external standards: caffeoyl quinic acid derivates using chlorogenic acid (Sigma-Aldrich, St. Louis, MO., U.S.A.), flavonoids with quercetin-3-rutinoside (Sigma-Aldrich) and sinapic acid derivatives using sinapinic acid (Sigma-Aldrich).

### Glucosinolates analysis by UPLC-MS/MS

The freeze-dried powder of the total leaves of all the genotypes was processed to determine their glucosinolates contents by UPLC-TripleQuad-MS/MS. Freeze-dried powder of leaf tissue (150 mg) was extracted as deswcribe above up to the centrifugation step. Extraction was performed using SPE Strata-X cartridges (33u Polymeric Strong Cation), following the instructions of the manufacturer (Phenomenex, Torrance, CA, USA). The cartridges were conditioned with 2 mL of MeOH and equilibrated with 2 mL of ultrapure water/formic acid (98:2, v/v). After this step, the samples were diluted in 2 mL of water/formic acid (98:2, v/v) and applied to the column, then washed with water/formic acid (98:2, v/v) and aspirated until dryness. The target analytes were eluted with 1 mL of MeOH/formic acid (98:2, v/v) and dried to completion using a SpeedVac concentrator (Savant SPD121P, Thermo Scientific, Massachusetts, USA). The extracts were reconstituted with 200 μL of solvents A/B (90:10, v:v) [solvent A: 13 mM ammonium acetate (pH 4 with acetic acid) and solvent B: acetonitrile:acetic acid (99.9:0.1, v:v)] and filtered through a 0.45-μm polyethersulphone filter (Millipore). Samples (20 μL) were analyzed by UPLC-MS/MS (Agilent Technologies, Waldbronn, Germany).

Chromatographic separation of sinigrin and allyl-isothiocyanate was carried out on a ZORBAX Eclipse Plus C-18 Rapid Resolution HD column (2.1 × 50 mm, 1.8 μm) (Agilent Technologies, Waldrom, Germany). The column temperatures were held at 10°C (left and right). The Multiple Reaction Monitoring (MRM) dynamic mode was used in the positive mode, assigning ±0.650 min as the™ interval time for each retention time and preferential transitions of the corresponding analytes. The dwell time was set at 30 ms. The flow-rate was 0.3 mL min^−1^, using the linear gradient scheme (t; %B): (0.0; 12), (0.2; 20), (1.0; 52), (2.5; 95), and (2.5; 12). The optimal ESI conditions for maximal detection of the analytes were: gas temperature, 225°C; sheath gas temperature, 350°C; capillary voltage, 3500 V; nozzle voltage, 1250 V; sheath gas flow, 12%; gas flow, 10; nebuliser, 40. The acquisition time was 2.5 min for each sample, with a post-run of 1.5 min for the column equilibration. The MS parameter fragmentor (ion optics capillary exit voltage) and collision energy were optimized for each compound, to generate the most-abundant product ions for the MRM mode. Data acquisition was performed using MassHunter software version B.04.00 (Agilent, Waldrom, Germany). The concentrations of glucosinolates were calculated from the area ratio of the ion peaks of the compounds to those of the corresponding standard curves (prepared fresh each day).

Thus, the results were obtained by using a sensitive, reproducible and robust UPLC-MS/MS method which improved the resolution of the peaks, with high reproducibility of the retention times and shorter analysis times (ca. 1/10) than regular HPLC-DAD-ESI-MSn methods.

### Plasma membrane protein purification from roots

Root plasma membranes were purified using the two-phase, aqueous-polymer technique described previously (Muries et al., 2011). The protein concentration of the PIP-enriched fraction was determined with the RC DC Protein Assay kit (BioRad), using BSA as standard.

### Gel electrophoresis and immunoblotting

Plasma membrane from the roots of Arabidopsis plants was isolated as previously described. Protein samples (10 μg per lane) were analyzed by 12% sodium dodecyl sulfate-polyacrylamide gel electrophoresis (SDS-PAGE) after denaturation at 56°C for 20 min in the presence of 2% (w/v) SDS and 100 mM DTT, in order to separate aquaporin dimers (Borgnia et al., 1999). The proteins were transferred to a PVDF membrane for 20 min at 15 V, in an electrophoretic transfer cell (Trans-Blot SD cell, BioRad), using Towbin transfer buffer (Towbin et al., 1979) with the addition of 0.05% SDS. After this, the membrane was blocked for 1 h at room temperature; in Tris-Buffered Saline (TBS) containing 2% (w/v) skimmed dry milk. Then, the membrane was incubated for 1 h at room temperature in a buffer containing TBS with 0.05% Tween 20, in the presence of an antibody (dilution 1:3000) raised against the first 42 N-terminus residues of Arabidopsis PIP1;1 (Kammerloher et al., 1994) (kindly provided by Prof. Dr. Schäffner) or against the 11 C-terminus residues of *Brassica oleracea* var. Italica PIP2 (Boursiac et al., 2005) (kindly provided by Prof. Dr. Santoni) (dilution 1:3000). Goat anti-rabbit IgG coupled to horseradish peroxidase was used as the secondary antibody (dilution 1:20000). A chemiluminescent signal was developed using the West-Pico, Super Signal substrate (Pierce). Immunoblots were performed on samples from three independent experiments. The intensity of each band was determined by a GS-800™ calibrated densitometer (Bio Rad, Barcelona, Spain). Background correction was performed by sampling membrane regions without spots and averaging their signal. The intensity of each band is the difference between the raw intensity of the protein of interest and the background. The scanned bands were normalized by calculating the ratio relative to the corresponding Coomassie-stained bands, thus ensuring that different intensities were not due to differences in the amount of protein loaded.

### Statistical analysis

The data were statistically analyzed, using the SPSS 17.0 software package (LEAD Technologies, Inc., Chicago, USA), by Tukey's test. Significant differences were determined at *P* < 0.05.

## Results

### Growth parameters and relative water content

Shoot and root biomass, length of primary and lateral roots, leaf area and percentage of seed germination were determined for the different genotypes with or without salt (Table 1). Under non-saline conditions, there were no statistically significant differences in shoot fresh (FW) and dry weights (DW) among genotypes. However, root FW and DW of the *myb28myb29* mutant were roughly halved in respect to wild type (WT) or the *myb28* mutant, in accordance with a shorter primary root length, as previously reported (Beekwilder et al., 2008). By contrast, salinity reduced shoot DW in all genotypes, as expected for plants with a diminished leaf area, but it did not affect, in the double mutant, either root DW or primary and lateral root lengths; the single mutant showed an increase in total lateral root length. In the WT, salt stress reduced the primary and lateral root lengths, while DW was maintained due to the increase in the number of lateral roots.

**Table 1:** **Shoot and root fresh weight (FW, g plant^−1^) and dry weight (DW, g plant^−1^), primary root length (cm), total lateral root (LR, cm) length, leaf area (cm^2^), and percentage germination of the** ***Arabidopsis thaliana*** **ecotype Col-0 wild type (WT), single** (***myb28***) **and double** (***myb28myb29***) **knockout mutants under non-saline (0 mM NaCl) and saline (100 mM NaCl) conditions**.

**NaCl (mM)**	**Genotype**	**FW (g plant^−1^)**		**DW (g plant^−1^)**		**Primary root length (cm)**	**Total LR length (cm)**	**Leaf area (cm^2^)**	**Percentage germination (%)**
		**Shoot**	**Root**	**Shoot**	**Root**				
	WT	0.40 ± 0.03a^a^	0.13 ± 0.02b	0.03 ± 210^−3^a	0.008 ± 110^−3^a	14.88 ± 0.46a	4.12 ± 0.38a	2.46 ± 0.11a	86.72 ± 2.43a
0	*myb28myb29*	0.31 ± 0.01a	0.07 ± 0.01c	0.02 ± 1.910^−3^ab	0.005 ± 810^−4^b	9.33 ± 1.16b	1.61 ± 0.24c	1.26 ± 0.20b	55.08 ± 0.75b
	*myb28*	0.38 ± 0.01a	0.12 ± 0.01b	0.03 ± 310^−3^a	0.008 ± 810^−4^a	13.61 ± 0.41a	2.03 ± 0.25c	1.80 ± 0.11ab	64.85 ± 1.25ab
	WT	0.34 ± 0.04a	0.18 ± 0.02a	0.02 ± 1.810^−3^b	0.009 ± 910^−4^a	9.95 ± 0.70b	2.63 ± 0.28b	1.47 ± 0.34b	
100	*myb28myb29*	0.17 ± 0.02b	0.07 ± 0.00c	0.01 ± 1.610^−3^c	0.006 ± 110^−3^b	9.75 ± 0.90b	2.12 ± 0.27c	0.94 ± 0.09c	
	*myb28*	0.33 ± 0.02a	0.15 ± 0.02ab	0.02 ± 2.710^−3^b	0.008 ± 210^−3^a	10.76 ± 0.45b	3.23 ± 0.50ab	1.22 ± 0.08b	

a *Different letters indicate statistical differences (Tukey, P < 0.05, n = 20 for each treatment)*.

Total lateral root length in the *myb28myb29* mutant was lower than in WT or the *myb28* mutant but it was not affected by the presence of salt. Seed germination was reduced in the *myb28myb29* mutant compared to WT and the single *myb28* mutant.

Leaf area was greatest in WT followed by the *myb28* mutant and smallest in the *myb28myb29* mutant. Salinity decreased leaf area in all genotypes, with the exception of the *myb28* mutant where it remained stable.

### Hydraulic conductance and whole plant transpiration

**Figure 1:**
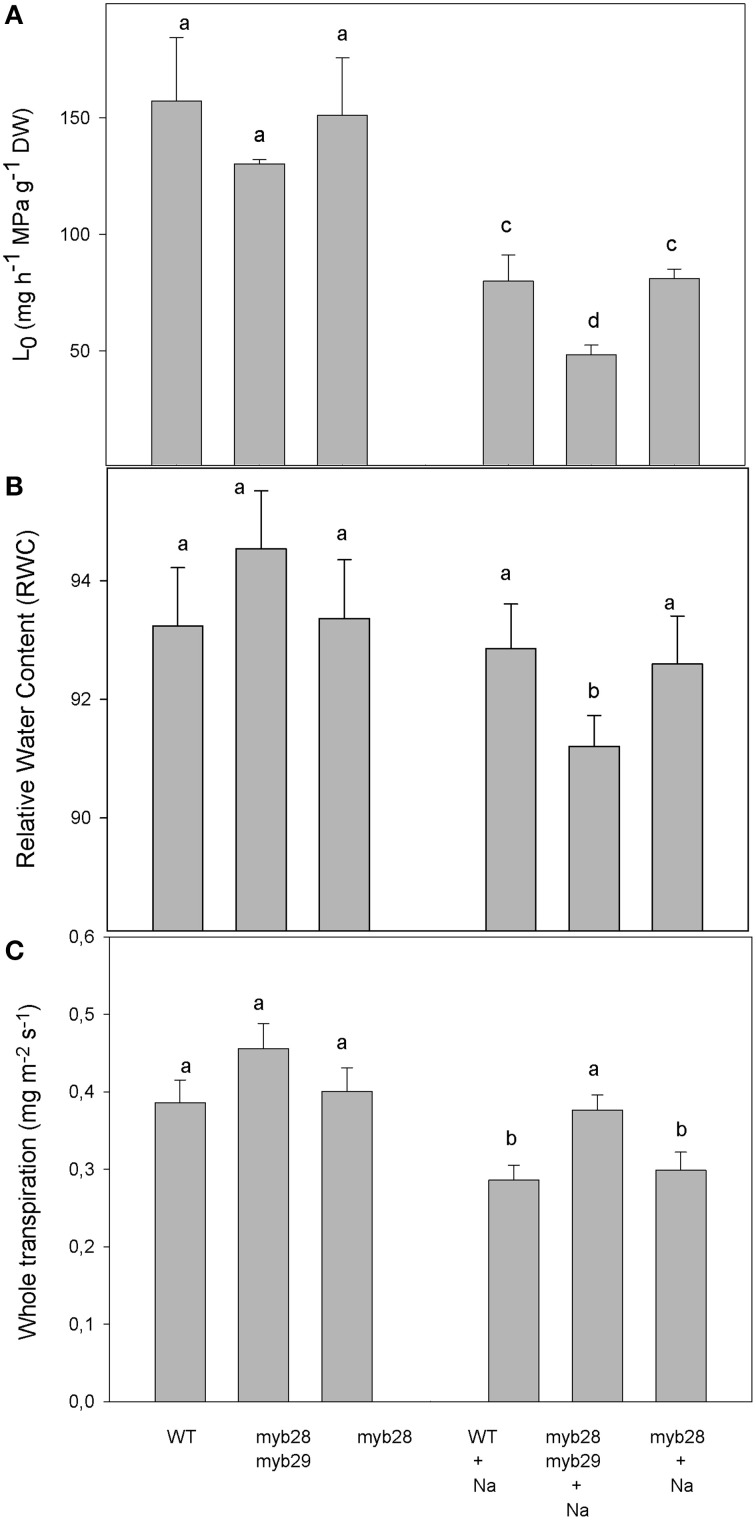
**Root hydraulic conductance, L_0_, (mg h^−1^ MPa^−1^ g^−1^ DW) (A), relative water content (RWC) (B) and whole plant transpiration (mg m^2^ s^−1^) (C) of** ***Arabidopsis thaliana*** **ecotype Col-0 wild type (WT), single** (***myb28***) **and double** (***myb28myb29***) **knockout mutants under non-saline (0 mM NaCl) and saline (100 mM NaCl) conditions**. Mean values ± standard errors are shown (*n* = 10). Mean values with different letters represent significant (*P* < 0.05) differences according to the Tukey test.

The root hydraulic conductance (L_0_) was determined for all three genotypes by root pressurization (Figure 1A). In the absence of the salt treatment, the L_0_ of the *myb28myb29* mutant was comparable to that of the WT and *myb28* mutant. Salinity reduced L_0_ by 49.1 and 46.3% in the WT and single mutant, respectively, and by 63.0% in the double mutant.

The relative water content (RWC) of the *myb28myb29* mutant was significantly reduced by NaCl addition, whereas salinity had no effect on the RWC in the WT and *myb28* mutant (Figure 1B).

Under salt stress, reductions in whole plant transpiration were higher in the WT and *myb28* mutant, which had similar values, compared to the *myb28myb29* mutant. However, under non-saline conditions, the whole plant transpiration was similar in all three genotypes (Figure 1C).

### PIP aquaporin abundance in the roots of the *Arabidopsis thaliana* genotypes

**Figure 2:**
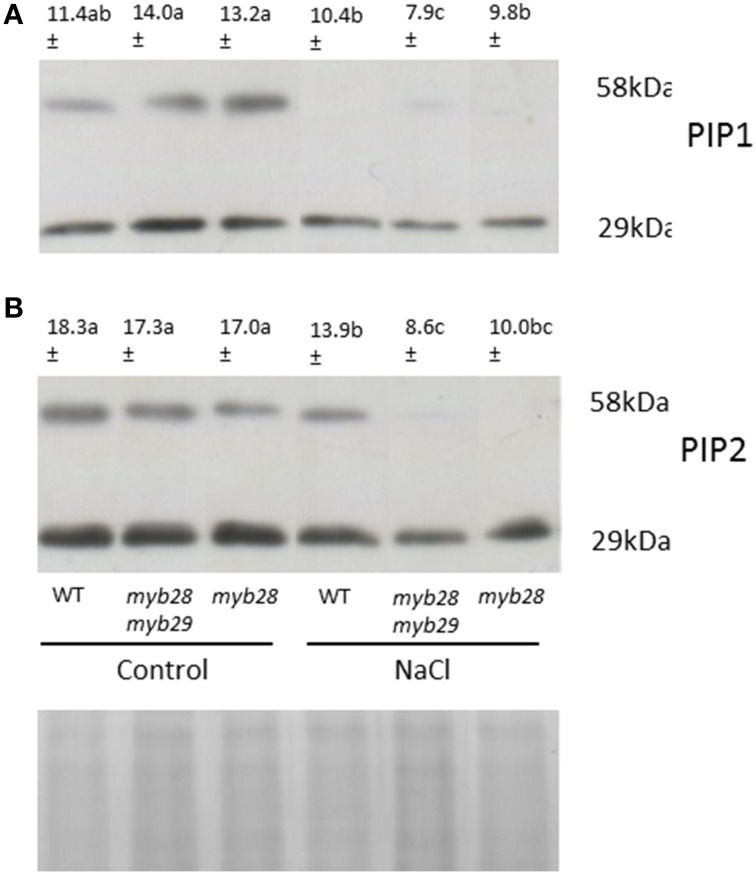
**Immunodetection of PIP1 (A) and PIP2 (B) homologs in the root plasma membrane (PM) of** ***Arabidopsis thaliana*** **ecotype Col-0 wild type (WT), single** (***myb28***) **and double** (***myb28myb29***) **knockout mutants under non-saline (0 mM NaCl) and saline (100 mM NaCl) conditions**. Total PM was separated by SDS-PAGE and probed with antibodies against *At*PIP1 and *At*PIP2. The PIP1 and PIP2 protein amounts (monomers and dimers) were quantified using the “Quantity one” program from Bio-Rad Laboratories. Mean values are shown (*n* = 3). Mean values with different letters represent significant (*P* < 0.05) differences according to the Tukey test. The scanned bands were normalized by calculating the ratio to the corresponding Coomassie-stained aquaporin band (29 KDa) ensuring that different intensities were not due to different loaded protein amounts.

For quantification of the *A. thaliana* PIP1 and PIP2 protein abundance in roots, Western Blotting was performed. Two specific antibodies developed against the PIP1 and PIP2 homologs of *A. thaliana* were used for protein immunodetection and two bands—at 29 kDa (monomeric form) and 58 kDa (dimeric form)—were detected in all samples (Figure 2). Under non-saline conditions, no significant differences were observed in the abundance of either PIP subfamily among the genotypes. However, the immunostain intensity differed among the genotypes when exposed to salinity. Thus, in general, the NaCl treatment decreased PIP1 (Figure 2A) and PIP2 (Figure 2B) abundance in the three genotypes, the reductions being 14.0, 56.4, and 21.2% in the PIP1 subfamily and 24.0, 50.3, and 41.2% in the PIP2 subfamily of the WT, *myb28myb29* and *myb28* plants, respectively.

Correlation between L_0_ and signal intensity of PIP2 and PIP2 was expressed in terms of the coefficient of determination (R^2^). R^2^ indicated the fraction of the variability in L_0_ that can be explained by the variability in aquaporin abundance through their linear relationship. High values for both PIP1 (*R*^2^ = 0.795) and PIP2 (*R*^2^ = 0.877) were observed.

### Mineral content

The ion concentrations in the leaves of the three genotypes were similar, under both non-saline and saline conditions (Table 2). Thus, salinity decreased the Ca, K, and S levels with regard to the control and increased the Na concentration in the three genotypes similarly, whereas the Mg, P, and N levels remained unchanged. The C level was increased by salinity in WT and *myb28* plants, but was unmodified in the *myb28myb29* mutant.

**Table 2:** **Calcium (Ca), potassium (K), magnesium (Mg), sodium (Na), phosphorus (P), sulfur (S), carbon (C), and nitrogen (N) (g 100 DW g^−1^) in the leaves of** ***Arabidopsis thaliana*** **ecotype Col-0 wild type (WT), single** (***myb28***) **and double** (***myb28myb29***) **knockout mutants under non-saline (0 mM NaCl) and saline (100 mM NaCl) conditions**.

**NaCl (mM)**	**Genotype**	**Macronutrients (g 100 DW g^−1^)**							
		**Ca**	**K**	**Mg**	**Na**	**P**	**S**	**C**	**N**
	WT	4.06 ± 0.43a	3.60 ± 0.35a	0.42 ± 0.06a	0.22 ± 0.05b	0.93 ± 0.06a	0.93 ± 0.06a	37.40 ± 0.26b	5.98 ± 0.52a
0	*myb28myb29*	3.45 ± 0.41a	3.74 ± 0.38a	0.38 ± 0.04a	0.19 ± 0.08b	0.88 ± 0.07a	0.941 ± 0.05a	38.42 ± 1.62b	5.59 ± 1.12a
	*myb28*	3.86 ± 0.33a	3.71 ± 0.37a	0.40 ± 0.04a	0.21 ± 0.05b	0.89 ± 0.06a	0.92 ± 0.03a	38.01 ± 0.56b	5.73 ± 0.49a
	WT	2.42 ± 0.14b	2.12 ± 0.26b	0.38 ± 0.04a	1.75 ± 0.15a	0.71 ± 0.08a	0.66 ± 0.03b	40.67 ± 0.75a	4.42 ± 0.49ab
100	*myb28myb29*	2.44 ± 0.22b	2.36 ± 0.26b	0.34 ± 0.03ab	1.65 ± 0.21a	0.78 ± 0.06a	0.67 ± 0.02b	38.86 ± 0.66b	5.65 ± 0.55a
	*myb28*	2.43 ± 0.19b	2.28 ± 0.21b	0.34 ± 0.03 a	1.70 ± 0.20a	0.73 ± 0.07a	0.66 ± 0.06b	40.32 ± 0.62a	4.72 ± 0.30ab

*^a^Different letters indicate statistical differences (Tukey, P < 0.05, n = 20 for each treatment)*.

### Glucosinolates and phenolic compounds

**Figure 3:**
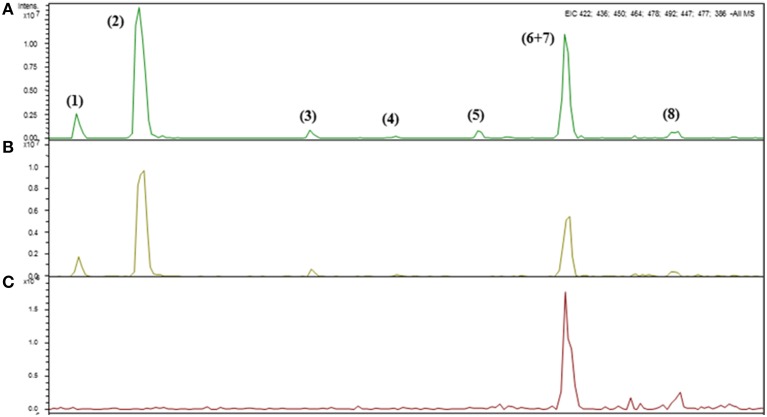
**Identification of intact glucosinolates by HPLC-DAD-ESI-MSn in the leaves of** ***Arabidopsis thaliana*** **ecotype Col-0 wild type (WT) (A), single** (***myb28***) **(B) and double** (***myb28myb29***) **(C) knockout mutants under non-saline (0 mM NaCl) conditions**.

The Arabidopsis leaves contained mainly glucoiberin (3MSOP), glucoraphanin (4MSOB), glucoalyssin (5MSOP), glucohesperin (6MSOH), glucosibarin (7MSOH) and glucohirsutin (8MSOO) as aliphatic glucosinolates (Table 3), while the major indole glucosinolates were glucobrassicin (I3M) and 4-methoxy-glucobrassicin (4MI3M), depending on the genotype. Thus, short-chain aliphatic glucosinolates were detected in the WT and *myb28* mutant, whereas long-chain aliphatic glucosinolates were found exclusively in the WT. The indole glucosinolates occurred in all three genotypes (Table 3, **Figure 3**).

**Table 3:** **ESI-MS data for glucosinolates detection in the different genotypes, abbreviation used in Figure 3 and retention time (RT)**.

**Ref.**	**Glucosinolate semisystematic name**	**Common name**	**RT (min)**	**[M–H]^−^**
1	3-methylsulphinylpropyl (3MSOP)	glucoiberin	4.6	422
2	4-methylsulphinylbutyl (4MSOB)	glucoraphanin	6.5	436
3	5-methylsulphinilpentyl (5MSOP)	glucoalyssin	11.6	450
4	6-methylsulphinylhexyl (6MSOH)	glucohesperin	14.2	464
5	7-methylsulphinylheptyl (7MSOH)	glucosibarin	16.7	478
6	3-indolylmethyl (I3M)	glucobrassicin	19.3	447
7	8-methylsulphinyloctyl (8MSOO)	glucohirsutin	19.3	492
8	4-methoxy-3-indolylmethyl (4MOI3M)	4-methoxyglucobrassicin	22.1	477

**Figure 4:**
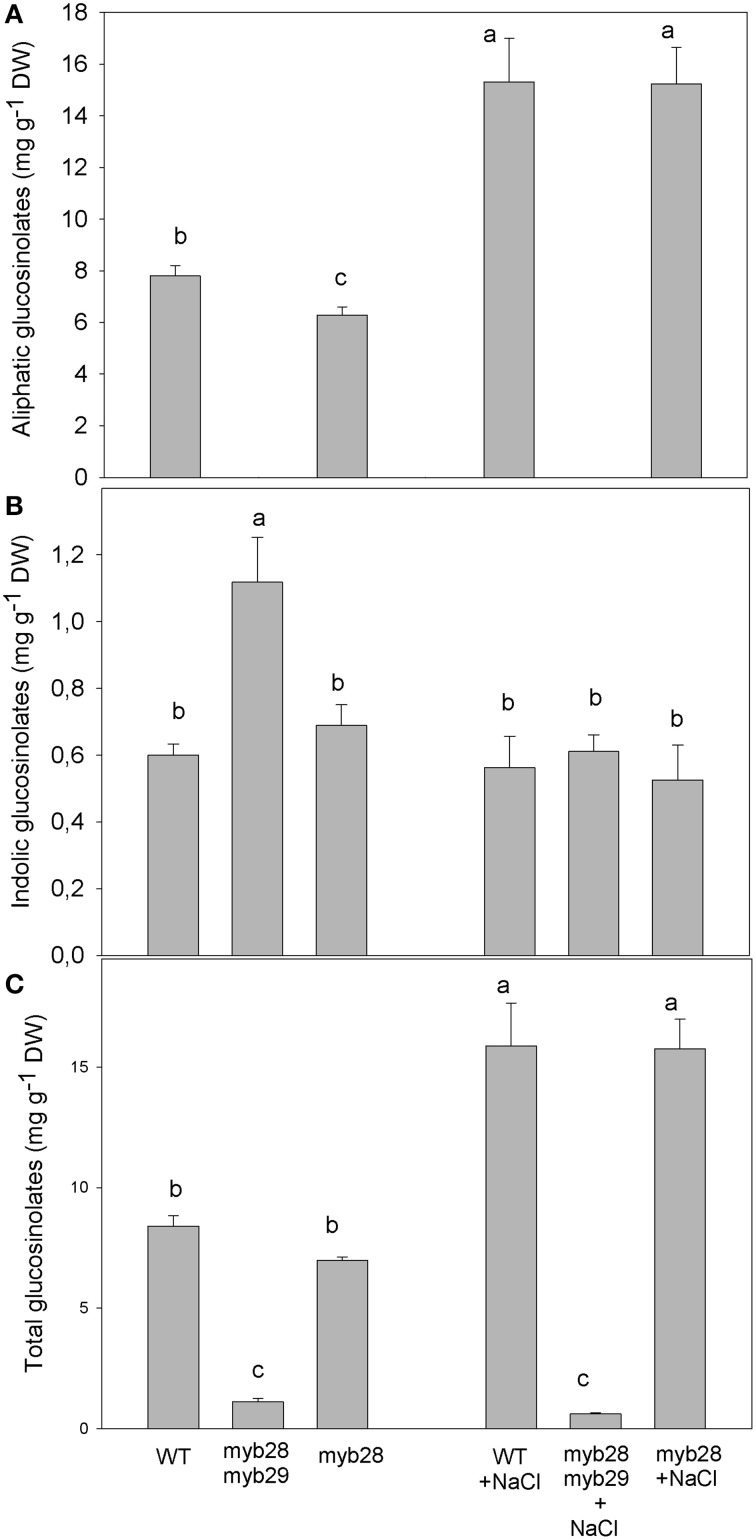
**Total aliphatic (A), total indolic (B) and total glucosinolates (C) (mg g^−1^ DW) in the leaves of** ***Arabidopsis thaliana*** **ecotype Col-0 wild type (WT), single** (***myb28***) **and double** (***myb28myb29***) **knockout mutants under non-saline (0 mM NaCl) and saline (100 mM NaCl) conditions**. Mean values ± standard errors are shown (*n* = 20). Mean values with different letters represent significant (*P* < 0.05) differences according to the Tukey test.

In general, aliphatic glucosinolates were increased by salinity in WT (~2-fold) and *myb28* mutant (~2.5-fold), reaching similar values (Figure 4). The level of the indolic glucosinolates was significantly higher in the *myb28myb29* mutant (~1.8-fold), compared to the WT and *myb28* mutant, but salinity decreased their content in the double mutant and left it unchanged in the WT and single mutant. Total glucosinolates were lower in the *myb28myb29* mutant and salinity had no effect on their content. By contrast, salinity caused similar increments (~2-fold) of total glucosinolates in the WT and *myb28 mutant*.

Regarding individual glucosinolates, salinity increased 3MSOP (~1.4-fold) and decreased 4MSOB (~1.2-fold) in the *myb28* mutant, whereas 5MSOP increased in the WT (~2.1-fold) and *myb28* mutant (~2.5-fold) (Table 4). The long-chain aliphatic glucosinolates 6MSOH, 7MSOH, and 8MSOO, detected only in the WT, were decreased by salinity, with 6MSOH becoming undetectable. The level of I3M was higher in the *myb28myb29* mutant, where it was significantly reduced by salinity (~11.5-fold). Similarly, in the *myb28* mutant, I3M was diminished by salt treatment (~1.3-fold), but it remained constant in WT. Under non-saline conditions, the 4MI3M content was similar in the three genotypes but salt stress decreased the level of this indolic glucosinolate in the *myb28myb29* (~2.5-fold) and *myb28* (~2.8-fold) mutants.

**Table 4:** **Individual glucosinolates in the leaves of** ***Arabidopsis thaliana*** **ecotype Col-0 wild type (WT), single** (***myb28***) **and double** (***myb28myb29***) **knockout mutants under non-saline (0 mM NaCl) and saline (100 mM NaCl) conditions**.

**NaCl (mM)**	**Genotype**	**Short-chain aliphatic glucosinolates**			**Long-chain aliphatic glucosinolates**			**Indolic glucosinolates**	
		**3MSOP**	**4MSOB**	**5MSOP**	**6MSOH**	**7MSOH**	**8MSOO**	**I3M**	**4MOI3M**
	WT	0.09 ± 0.007a	0.67 ± 0.045a	6.90 ± 0.358b	0.01 ± 0.001a	0.01 ± 0.001a	0.10 ± 0.008a	0.54 ± 0.030c	0.06 ± 0.004a
0	myb28myb29	n.d	n.d	n.d	n.d	n.d	n.d	1.06 ± 0.127a	0.06 ± 0.008a
	myb28	0.03 ± 0.002c	0.49 ± 0.051b	5.77 ± 0.389b	n.d	n.d	n.d	0.69 ± 0.049b	0.06 ± 0.007a
	WT	0.10 ± 0.017a	0.61 ± 0.104a	14.52 ± 3.136a	n.d	0.005 ± 0.001b	0.06 ± 0.011b	0.50 ± 0.086c	0.06 ± 0.007a
100	myb28myb29	n.d	n.d	n.d	n.d	n.d	n.d	0.09 ± 0.052d	0.02 ± 0.002b
	myb28	0.04 ± 0.003b	0.40 ± 0.043c	14.79 ± 1.142a	n.d	n.d	n.d	0.53 ± 0.068c	0.02 ± 0.002b

*Short-chain aliphatic glucosinolates (3MSOP, 4MSOB, and 5MSOP), long-chain aliphatic glucosinolates (6MSOH, 7MSOH, and 8MSOO) and indolic glucosinolates (I3M, 4MOI3M) were detected*.

*^a^Different letters indicate statistical differences (Tukey, P < 0.05, n = 20 for each treatment)*.

**Figure 5:**
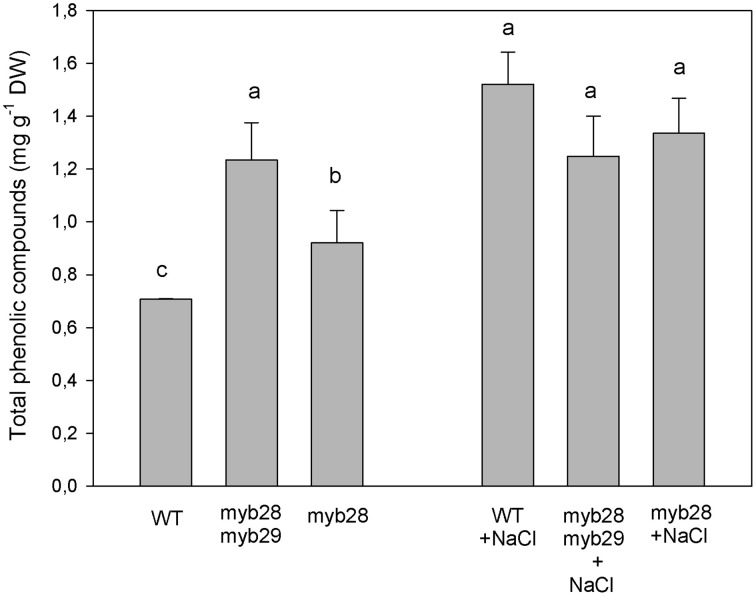
**Total phenolic compounds (mg g^−1^ DW), as the sum of chlorogenic acid, flavonoids and sinapic acid derivates, in the leaves of** ***Arabidopsis thaliana*** **ecotype Col-0 wild type (WT), single** (***myb28***) **and double** (***myb28myb29***) **knockout mutants under non-saline (0 mM NaCl) and saline (100 mM NaCl) conditions**. Mean values ± standard errors are shown (*n* = 20). Mean values with different letters represent significant (*P* < 0.05) differences according to the Tukey test.

The total phenolic compounds, (defined as the sum of chlorogenic acid, flavonoids, and sinapic acid derivates), in the different genotypes are presented in Figure 5: under non-saline conditions, they were highest in the *myb28myb29* mutant, followed by *myb28* and the WT. Salinity increased the levels of phenolic compounds in the WT and *myb28* mutant.

## Discussion

Previous analysis of the *myb28* and *myb29* single and *myb28myb29* double knockout-mutant lines demonstrated that these two *Myb* genes cooperate to regulate aliphatic glucosinolates biosynthesis and that the presence of at least one functional gene is necessary for gene expression and metabolite accumulation. Not surprisingly modulation of the glucosinolate content influenced the extent of plant damage caused by the larvae of the lepidopteran insect *Mamestra brassicae* (Beekwilder et al., 2008). We explore in this study the effect of varying aliphatic and indolic glucosinolate level by genetic means on the response of these plants to abiotic stress. In particular, the connection between glucosinolates and water uptake and transport under salinity has been explored. As reported previously by Beekwilder et al. (2008), the initial growth of the double knockout line was delayed, and we provide some interpretation of this effect. We found that the *myb28myb29* mutant had lower leaf area and root biomass than the WT under non-saline conditions, due to its reduced primary and lateral root (LR) lengths at the developmental stage investigated. In previous work, we observed an increase of glucosinolates at the highest salt concentration in relation to stress resistance and growth (Lopez-Berenguer et al., 2008) indicating that glucosinolates could be an adaptive component of salt tolerance (Qasim et al., 2003). These results have been related to the aquaporins function due to their role in root water uptake. Thus, aquaporins and hydraulic conductivity seem to be associated to changes in the anatomical and morphological features of the roots affecting nutritional status (Martínez-Ballesta et al., 2011). Therefore, the most parsimonious interpretation of our data is that the decrease in aliphatic glucosinolates expression and/or the increase in indolic glucosinolates, modify aquaporin abundance. This is consonant with a previous report where the lack of long-chain aliphatic glucosinolates in the *cyp79F2* mutants of *A. thaliana* reduced LRs growth (Tantikanjana et al., 2004).

Leaf area and root hydraulic conductance under salt stress would then be affected primarily by root morphology. A decrease in the root hydraulic conductance (L_0_) may lead to leaf area reductions as a mechanism of plant response to salt stress. In this situation, the control of L_0_ mediated by root aquaporins may be crucial - as in the *myb28myb29* mutant, where L_0_ was not synchronized with stomatal behavior and transpiration was unmodified.

Also, salt stress may induce morphological changes in the roots of *A. thaliana*; such as inhibition of primary root elongation and simultaneous increases in the LR number and branching under moderate salt stress (Potters et al., 2007; Zolla et al., 2010). These results agree with the morphological root changes observed in the WT, whereas increased LRs length in the single mutant may compensate the lack of more LRs. In Arabidopsis, salt stress can induce a strong reduction in the number of lateral root primordia (LRP) (Burssens et al., 2000) or, by contrast, an increase in LR number; for example, in Arabidopsis, *Cicer arietinum* and *Oryza sativa* cultivars (He et al., 2005; Shukla et al., 2006; Nibau et al., 2008; Krishnamurthy et al., 2011). A clear genotypic effect on the response of the root system to salinity is shown in this work. Salinity had no effect on the primary and lateral root lengths in the double mutant. The intrinsic reduced root system in this genotype, where there was a complete absence of aliphatic glucosinolates could originate that salinity did not modify root architecture. By contrast, the highest increase in the aliphatic glucosinolates content under salt stress was in the *myb28* genotype, relative to the WT, and this coincided with stimulation of LR growth.

The relationship between aliphatic/indolic glucosinolates and root proliferation under salt stress needs further research. In addition, the percentage seed germination declined as the aliphatic glucosinolate levels decreased, both being lower in the *myb28myb29* mutant, and as the levels of indolic glucosinolates increased. The effect could be again mediated by alterations in auxin content or sensing or by breakdown products of glucosinolates which contribute to broccoli seed germination, for instance as sulfur donors during germination (Gao et al., 2014).

The mineral content was unaffected by the mutations, except for carbon. The absence of aliphatic glucosinolates resulted in unmodified, steady-state transpiration in the *myb28myb29* genotype under salt stress; this may have maintained the uptake of ions and could explain the lack of difference in their tissue accumulation in relation to the WT. Moreover, a lower RWC may have concentrated the foliar ions in salt treated *myb28myb29* plants. The modest carbon increase in WT and *myb28* mutant under salinity was probably related to increased accumulation of carbohydrates or other carbon rich compounds, a strategy to cope with salt stress. In fact, sugars, which accumulate in response to environmental stresses, may act as osmolytes to maintain cell turgor and they have been previously shown to increase in Arabidopsis plants under salt stress (Krasensky and Jonak, 2012). This response may not occur in the double mutant.

The good correlation between L_0_ and the protein levels of both PIP1 (*R*^2^ = 0.795) and PIP2 (*R*^2^ = 0.877) suggest a symplastic regulation of water flow under both non-saline and saline conditions. Aquaporin abundance in root plasma membrane may have contributed to the differences in L_0_- among genotypes. It has been reported previously that perturbation of glucosinolate biosynthesis by suppression of the *CYP79F1* and *CYP79F2* genes, (by RNAi), modified the expression of cell environment-interacting proteins (by ~12%) among others, including PIP2a (Chen et al., 2012). Similar correlation between L_0_ and the PIP1 content was observed in broccoli plants after exogenous sinigrin addition and the subsequent allyl-isothiocyanate formation. However, the PIP2 content was not related to the L_0_ values under the above conditions, although under salt stress the greater sinigrin effect on osmotic water permeability (P_f_)—rather than L_0_—provided evidence for the contribution of PIP2 aquaporins to the cell-to-cell pathway (Martínez-Ballesta et al., 2014). Differences among exogenous and endogenous glucosinolates, genotypes and stress conditions may influence the contribution of aquaporins to this route.

Under non-saline conditions, in spite of the phenotypic alteration shown by *myb28myb29* plants (reduced leaf and root growth), plant water relations were comparable with L_0_ contributions in all genotypes. However, under salinity, the greater reduction in L_0_ in the double mutant plants, together with the maintenance of whole plant transpiration, reduced their RWC. These results show that the contribution of short-chain aliphatic glucosinolates to water saving are more important under natural conditions—whereas variations in the environment, such as salt stress, may require an adjustment of stomatal opening. It has been shown previously that addition of glucosinolate breakdown product, for instance allyl-isothiocyanate, under salt stress increased the abundance of the PIP2 subfamily and led to the recovery of L_0_, with respect to the addition of NaCl alone (Martínez-Ballesta et al., 2014). This positive effect of glucosinolate byproducts in the face of salinity could help to explain the fact that the lack of aliphatic glucosinolates in the *myb28myb29* genotype and the related PIP2 abundance resulted in a lower L_0_ under salt stress. Furthermore, under salt stress, axial resistance to water transport may contribute to total root resistance (Passioura, 1977; Rewald et al., 2011c) and root branching could influence L_0_ (Joly, 1989). The increased LRs number in the WT and LRs length in the *myb28* mutant under salt stress may account for their similar L_0_ values, in addition to the lesser reduction in PIPs abundance, compared to the *myb28myb29* mutant.

The transcription factors MYB28 and MYB29, together with MYB76, are the main regulators of aliphatic glucosinolate biosynthesis (Gigolashvili et al., 2007a,b; Hirai et al., 2007). In the double mutant, all aliphatic glucosinolates (long- or short-chain) were below the detection level, even in sensitive LC-MS analyses. Coordinated control of different glucosinolate biosynthetic pathways has been observed and the inhibition of one class of glucosinolates resulted in a compensatory increase in another class (Hemm et al., 2003; Grubb and Abel, 2006; Yan and Chen, 2007; Beekwilder et al., 2008; Malitsky et al., 2008). Thus, in our experiments indolic glucosinolates increased in the *myb2829* mutants, compensating the lack of aliphatic glucosinolates—as also found in previous studies (Hirai et al., 2007; Beekwilder et al., 2008). Under salt stress, aliphatic glucosinolates increased in WT and *myb28*. This is related to the aquaporins expression as it has been suggested by their involvement on osmoregulation pathways (Martínez-Ballesta et al., 2014): this may have led to the improved water status of WT and *myb28* plants under stress. Previous findings indicated that salt-induced increases in the glucosinolate content may be involved in the salt-stress response of the halophyte *Thellungiella salsuginea* (Pang et al., 2009, 2012). However, variations in the individual glucosinolates and particular developmental stages were considered. Thus, the link between PIP abundance, root hydraulic conductance and the presence of a particular aliphatic glucosinolate in glycophytes is now well stated and deserves further attention, especially in crops and where the induction of an individual glucosinolate is involved in increasing resistance to salt stress.

## Conclusion

The results presented suggest that the pathways involved in the physiological responses to salt stress are connected to glucosinolate metabolism (i.e., the production of isothiocyanates and indoles) which is influenced by abiotic stress factors. In this sense, the root and leaf morphological changes provoked by salinity showed genotypic dependence and aliphatic, rather than indole, glucosinolates may condition these responses. Given the link between the L_0_ and PIP aquaporins, one would expect their involvement in the observed change in L_0_, indicating the contribution of these proteins to water flow through the root. The simplest interpretation for the differences in growth and responses to salt stress among the three genotypes could invoke differences in auxin content or sensing, however the lack of short-chain aliphatic glucosinolates or their hydrolysis products may affect more directly plant water relations and aquaporins response under salt stress. Future, research will need to address how internal and external factors that regulate glucosinolate metabolism affect planta and to identify which signaling molecules are involved in the response to salt stress and the mechanisms of the interconnected glucosinolate-aquaporin relation. A first indication of water balance adaptation to salinity influenced by the availability of aliphatic glucosinolates as elements involved in plant osmoregulation is given. In addition, whether the perturbation of aliphatic glucosinolates is sufficient to affect auxin synthesis in the shoot (and therefore root morphology) could be obtained by crossing the double knockout line to the DR5:GUS reporter in order to visualize auxin concentration in the mutant.

### Conflict of interest statement

The authors declare that the research was conducted in the absence of any commercial or financial relationships that could be construed as a potential conflict of interest.

## Abbreviations

BSA: bovine serum albumin
DAD: Diode Array Detector
ESI: Electrospray interface
SDS-PAGE: sodium dodecyl sulfate-polyacrylamide gel electrophoresis.
